# Date-Palm-Derived Cellulose Nanocrystals as Reinforcing Agents for Poly(vinyl alcohol)/Guar-Gum-Based Phase-Separated Composite Films

**DOI:** 10.3390/nano12071104

**Published:** 2022-03-27

**Authors:** Hamid M. Shaikh, Arfat Anis, Anesh Manjaly Poulose, Niyaz Ahamad Madhar, Saeed M. Al-Zahrani

**Affiliations:** 1 SABIC Polymer Research Centre, Department of Chemical Engineering, King Saud University, P.O. Box 800, Riyadh 11421, Saudi Arabia; aarfat@ksu.edu.sa (A.A.); apoulose@ksu.edu.sa (A.M.P.); szahrani@ksu.edu.sa (S.M.A.-Z.); 2 Department of Physics and Astronomy, College of Sciences, King Saud University, P.O. Box 2455, Riyadh 11451, Saudi Arabia; nmadhar@ksu.edu.sa

**Keywords:** date palm, cellulose nanocrystals, poly(vinyl alcohol), guar gum, phase-separated films, moxifloxacin, drug delivery

## Abstract

The current study delineates the use of date-palm-derived cellulose nanocrystals (dp-CNCs) as reinforcing agents. dp-CNCs were incorporated in varying amounts to poly(vinyl alcohol)/guar-gum-based phase-separated composite films. The films were prepared by using the solution casting method, which employed glutaraldehyde as the crosslinking agent. Subsequently, the films were characterized by bright field and polarizing microscopy, UV-Vis spectroscopy, FTIR spectroscopy, and mechanical study. The microscopic techniques suggested that phase-separated films were formed, whose microstructure could be tailored by incorporating dp-CNCs. At higher levels of dp-CNC content, microcracks could be observed in the films. The transparency of the phase-separated films was not significantly altered when the dp-CNC content was on the lower side. FTIR spectroscopy suggested the presence of hydrogen bonding within the phase-separated films. dp-CNCs showed reinforcing effects at the lowest amount, whereas the mechanical properties of the films were compromised at higher dp-CNC content. Moxifloxacin was included in the films to determine the capability of the films as a drug delivery vehicle. It was found that the release of the drug could be tailored by altering the dp-CNC content within the phase-separated films. In gist, the developed dp-CNC-loaded poly(vinyl alcohol)/guar-gum-based phase-separated composite films could be explored as a drug delivery vehicle.

## 1. Introduction

Recently, PVA- and polysaccharide-based phase-separated films have been proposed by several authors. Phase-separated systems are a special type of composite system wherein the different phases are formed due to the thermodynamic incompatibility between the two polymeric phases. Yadav et al. (2017) have synthesized PVA and carboxymethyl tamarind gum-based composite films [1]. Herein, the dispersed phase was carboxymethyl tamarind gum, while the PVA formed the continuum matrices. The films were reported to form phase-separated structures. The authors noted that the carboxymethyl tamarind gum-containing composite films supported better human keratinocyte proliferation. The composite films were also found to be capable of antimicrobial drug delivery applications. In another study, the same group further reported that the properties of the composite films were significantly changed when the internal carboxymethyl tamarind gum phase was reinforced with graphene oxide nanosheets [2]. The blank films showed good antimicrobial properties, which was accounted for the antimicrobial activity of graphene oxide nanosheets. The films were also explored for antimicrobial drug delivery applications. It was found that the films were biocompatible towards human keratinocytes.

Polyvinyl alcohol (PVA) is a semi-crystalline polymer [3]. The polymer is highly hydrophilic, due to which it can be easily soluble in water and is an excellent film-forming polymer [4]. It has been extensively used to develop polymeric matrices for wide applications, including drug delivery, tissue engineering, regenerative medicine, food packaging, and sensor development [5,6]. The biological applications of PVA are motivated because of its excellent biocompatibility with human tissue [7]. Nevertheless, the polymer matrices of PVA have been reported to exhibit poor mechanical and thermal stability. As mentioned previously, the hydrophilic nature of PVA results in quick water absorption when placed in the biological environment [8]. The absorption of water molecules results in the mechanical instability of the PVA matrices, thereby leading to their disruption. The polymer matrices of PVA have been crosslinked either by physical or chemical methods for improving mechanical stability even when such matrices are placed in an aqueous environment [9]. Further, various authors have reported the inclusion of reinforcing materials (e.g., nanocellulose, carbon nanotubes, and their derivatives, silver nanoparticles, etc.) for enhancing the mechanical stability of PVA [10,11,12].

Similarly, many researchers have proposed the synthesis of polymeric architectures by blending PVA and other biological polymers [13,14]. The blending of polymers of biological origin with PVA allows the researchers to modulate the functionality of the polymeric architectures. For example, polymeric structures of PVA and gelatin, alginate, chitosan, or pectin have been proposed for drug delivery and tissue engineering applications [15,16,17,18]. Recently, PVA and polysaccharide-based phase-separated films have been proposed by several authors. In such systems, the polysaccharides from the dispersed phase. The main advantage of such a system can be related to the reinforcing effect exerted by the polysaccharide phase, which helps to improve the mechanical stability of the films, compared with the pristine PVA film. However, the mechanical stability of such phase-separated films is composition-dependent. It has been found that, at a critical concentration, the mechanical stability of the phase-separated film is the highest. The alteration in mechanical properties can be related to the microstructural arrangement and the crystallinity of the polymeric phases.

Guar gum (GG) is a widely used polysaccharide-based biopolymer that is extracted from the embryos of *Cyamopsis tetragonoloba* [19]. Chemically, GG has a linear backbone of (1–4)-β-D-mannopyranosyl units, which consists of α-D-galactopyranosyl units as pendants. The gum has been proposed as a coating material for site-specific drug delivery [20]. Similarly, cellulose is another one of the most abundant naturally occurring polysaccharides from plant cell walls in which a glucose unit of β-1,4 D-glucopyranosyl (anhydroglucose (AGU)) is joined linearly in the ^4^C_1_-chain configuration. The high degree of polymerization, high surface-area-to-volume ratio, and availability of numerous chemical functional groups in the nanocellulose offer a high loading and binding capacity for drug release [21]. These polysaccharides are extensively used as biomaterials due to their biodegradable and biocompatible characteristics.

Nonetheless, cellulose nanocrystals (CNCs) are also derived from agricultural waste materials. Valorization of the CNCs has been reported to develop several polymeric architectures for various applications, including photonic, pharmaceutical, and biomedical applications. The dp-CNCs are being synthesized from the wastes of the date palm industry in our previous research [22]. Moreover, the reinforcing effect of the dp-CNCs on the phase-separated composite films has not been studied yet. Hence, examining the ability of the dp-CNCs in tailoring the properties of phase-separated composite films seems rather justified.

Therefore, in this study, we propose to develop PVA–guar gum (GG) phase-separated film where the GG would form the dispersed phase, and the PVA would form the external phase. The PVA–GG film would be reinforced with date-palm-derived cellulose nanocrystals (dp-CNCs). A thorough literature survey suggests that composite films of such compositions have not been investigated. Accordingly, PVA–GG (PGC) phase-separated films reinforced with dp-CNCs are fabricated using a solvent casting method, and its structure–property relationship for drug delivery analysis is studied in this study.

## 2. Materials and Methods

### 2.1. Materials

Polyvinyl alcohol (Mw 89,000–98,000, 99+% hydrolyzed CAS number 9002-89-5), glutaraldehyde solution 25% (*v*/*v*), were purchased from Sigma Aldrich, St. Louis, MO, USA. Isopropanol (99.9%, analytical grade), and hydrochloric acid (37%, ACS reagent) were obtained from Panreac Química, Garraf, Spain. Guar gum was supplied by Scharlab, Barcelona, Spain. dp-CNCs were synthesized in our lab, as per the method mentioned earlier. In brief, fine powder was obtained from palm tree trunk mesh and pretreated with supercritical carbon dioxide (ScCO_2_) to eliminate the water-soluble extractives. It was then treated with a 20% (*w*/*v*) sodium hydroxide solution at 90 °C for 6 h. The solid fraction was separated and washed until it became alkali-free. Finally, a bleaching reaction was carried out at 70 °C for 4 h, using an acidic solution of sodium chloride (pH 3.7). The pure cellulose was collected by filtration, washed several times until it became neutral, and finally dried to obtain a constant weight. For obtaining nanocellulose from this cellulose, a combination of mechanical disintegration and chemical treatments with sulfuric acid was used. Firstly, homogenous cellulose suspension has a solid content of ~5 wt.%. The suspension was then fed into the barrel of a twin-screw DSM-Xplore micro-compounder (15 cm^3^ Xplore^®^, Sittard, The Netherland) for mechanical defibrillation of cellulose. It is continuously recirculated within a barrel for about 30 min at a constant screw speed of 250 rpm. After this treatment, solids were collected and subjected to sulfuric acid hydrolysis. In short, 10 g treated cellulose samples were hydrolyzed in 100 mL 50 wt.% H_2_SO_4_ solution. The reaction was performed at 45 °C with continuous stirring for about 60 min. Finally, the hydrolysis reaction was quenched by the addition of a large amount of distilled water. This suspension was centrifuged several times, and the supernatant fluid was discarded till it became neutral. This cloudy suspension was then dialyzed against distilled water until the pH of the suspension reached a constant value. This portion of the nanocellulose suspension was stored in a refrigerator at 4 °C, while the other was freeze-dried and utilized for further use. Furthermore, it has nanoparticles with sizes ranging from 26 nm to 61 nm, a negative zeta potential of −35 mV, and 89% crystallinity [22]. Furthermore, double distilled water was used throughout the study.

### 2.2. Preparation of Nanocomposite Films

**Figure 1:**
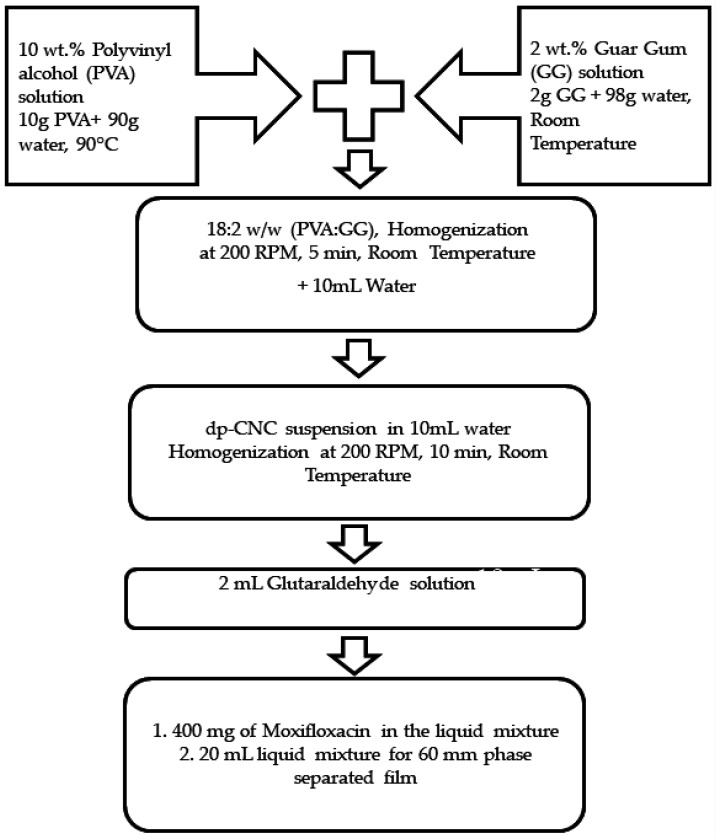
Flowchart diagram of the preparation of the films.

The PVA–GG-based nanocomposite films were prepared similar to the method proposed by Yadav et al. [2]. In short, initially, solutions of 10% (*w*/*w*) PVA and 2% (*w*/*w*) GG in water were prepared. To make a 10% aqueous solution of PVA, 10 g of PVA was gradually dispersed in 90 g of water by stirring. The mixture was then put in a water bath at 90 °C to achieve complete dissolution of PVA. For GG preparation, 2 wt.% GG was prepared at room temperature by dissolving 2 g GC in 98 g of hot water. Then, the solutions of PVA and GG were mixed in the ratio of 18:2 (*w*/*w*) and homogenized at room temperature using an overhead stirrer (200 rpm; 5 min). A volume of 10 mL of water was then added to the mixture and subsequently homogenized. A suspension of dp-CNC in 10 mL water, containing 0 mg, 2.5 mg, 5 mg, 7.5 mg, and 10 mg, was then added to the diluted solution and homogenized further for another 5 min. This was followed by the addition of the 2 mL of glutaraldehyde reagent, which was used as the crosslinking solution. The glutaraldehyde reagent was prepared by mixing glutaraldehyde, isopropanol, and hydrochloric acid in the ratio of 0.5:0.5:0.05 (i.e., 0.5 mL of glutaraldehyde, 0.5 mL of isopropanol, and 0.05 mL of hydrochloric acid). The liquid mixtures (42 mL) were then converted into films of 60 mm diameter by the solution casting method using 20 mL of the mixtures. During the drying stage, the liquid mixtures, which were poured into Petri dishes, were kept in a thermal cabinet (40 °C) for 24 h. The drying process resulted in the formation of films, which were peeled off with the help of forceps. The films were named A0, A1, A2, A3, and A4, respectively. A0 film was a phase-separated film of PVA and GG, devoid of dp-CNC reinforcement. The rest of the films contained 2.5 mg, 5 mg, 7.5 mg, and 10 mg of dp-CNCs, respectively, in the 40 mL of PVA–GG mixture. The drug-loaded films were synthesized by incorporating 400 mg of moxifloxacin in the liquid mixture after the addition of the crosslinking agent. The films thus formed were named A0D, A1D, A2D, A3D, and A4D. Figure 1 illustrates the flowchart of the film formulation.

### 2.3. Characterization of the Films

#### 2.3.1. Microstructure Analysis

The microstructures of the films were observed and analyzed under bright-field (Model: DM750, Leica Microsystems, Wetzlar, Germany) and polarizing (Model DM 75, Leica Microsystems, Wetzlar, Germany) microscopes.

#### 2.3.2. Spectroscopic Analyses

The UV-Vis transmission spectrum of the films was evaluated using a UV-Vis spectroscope (Shimadzu 3600 UV-VIS-NIR, Kyoto, Japan). The scanning was carried out in the wavelength region of 280 nm and 800 nm. The functional group analysis and the interactions among the functional groups were analyzed using a Fourier transform infrared (FTIR) spectroscope (Nicolet iN10, Thermo Scientific, Winsford, UK). The measurements were made in the attenuated total reflectance (ATR) mode at room temperature.

#### 2.3.3. X-ray Diffraction Study

The films were subjected to wide-angle X-ray diffraction studies using an X-ray diffractometer (D8 Advance, Bruker, Berlin, Germany). An automated wide-angle goniometer coupled with a sealed tube with Cu-Kα source radiation (λ = 1.54056 Å) was used. In reflection mode, a range of 2θ was scanned from 5° to 50° at 5°/min, and the X-ray tube was operated at 40 kV and 40 mA.

#### 2.3.4. Mechanical Study

The mechanical property of the films was estimated by performing the stress relaxation (SR) study, which can divulge information about the viscoelastic nature of the films. The samples were cut into rectangular pieces (size (L × B): 60 mm × 5 mm). Then, the film pieces were attached to the sample holder and placed under the tensile grip. The length of the sample holder window was maintained at 50 mm. The test was carried out by stretching the films by 2 mm and then recording the reduction in the stress values for 60 s. The analysis was carried out in triplicate.

#### 2.3.5. Drug Release Study

The drug release study was conducted in Franz’s diffusion cell. The receptor compartment of the diffusion cell was 12.0 mL. The films were cut into circular pieces (1 cm diameter) so that the drug content in the films was 1.57 mg/cm^2^. The receptor compartment was filled with phosphate-buffered saline (PBS; 6.8). Then, an activated dialysis tube was placed over the receptor compartment, followed by the placement of the films. Thereafter, the donor compartment was placed and secured, followed by the addition of 1.0 mL of PBS. The PBS in the receptor compartment was sampled (1.00 mL) at regular intervals, which was then replaced with fresh PBS. The sampled PBS was then analyzed in a UV-Vis spectrometer at 391 nm wavelength to determine the drug content. The release study was conducted in triplicate.

#### 2.3.6. Statistical Analysis

The results of mechanical and drug release studies are reported as average ± standard deviation. The variation in the average values was analyzed by *t*-test.

## 3. Results and Discussions

### 3.1. Microstructure Analysis

**Figure 2:**
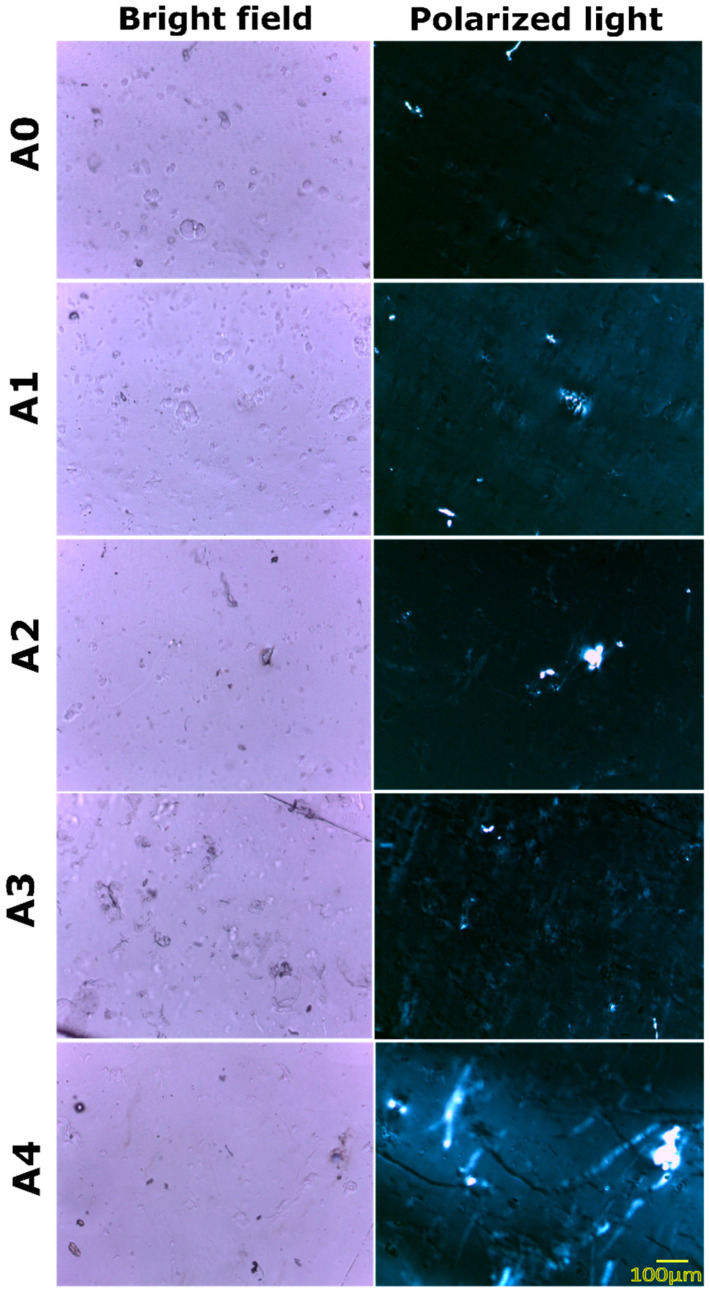
Bright-field and polarizing micrographs of the prepared films.

The addition of dp-CNCs in A1, A2, A3, and A4 was expected to alter the microstructure of the films considerably. The bright-field micrographs (Figure 2) of A1, A2, and A3 showed the presence of the dispersed phases. The agglomeration of the dispersed phase of GG was found to be composition-dependent. Interestingly, it was found that the microarchitecture of A4 was relatively smoother than the other films. Such an observation can only be explained by the ability of dp-CNCs to reduce the interfacial tension among PVA and GG molecules when their concentrations were highest. The bright-field micrographs of A0 film (control), which did not contain dp-CNCs, showed the presence of dispersed phases. This observation was in concurrence with the observations made by Yadav et al. [2], wherein the formation of phase-separated films of PVA was reported. 

The polarizing micrographs of A0 also showed the presence of these phase-separated structures. However, the overall brightness of the polarizing micrograph was relatively dark. The dark appearance of the micrographs was due to the positioning of the polarizer and the analyzer in the cross-polarized position. The polarized light micrographs of A1, A2, and A3 confirmed the presence of GG as the dispersed phases. The microarchitecture of A3 showed agglomerated structures of the dispersed phase. The extent of the agglomerated structures was relatively greater in A3 as against A1 and A2, which predominantly showed isolated dispersed phases. Interestingly, the polarized light micrographs of A3 also showed some minor microcracks within the film structure. The polarized light micrographs of A4 showed the extensive presence of microcracks, which were more predominant than the microcracks of A3.

Moreover, the prepared films were colorless and transparent (Figure S1). The formation of colorless and transparent films of PVA and polysaccharides has been reported earlier by several research groups [2]. Such an observation can be related to the presence of PVA, which forms colorless and transparent films, in a higher amount than the polysaccharide. The prepared films were firm to touch and could be appropriately handled without damaging their structure. Apart from this, the foldability of the films was also very good. This can be reasoned to the presence of PVA as the continuum matrix. The films were placed over a scale to judge their apparent transparency (Supplementary Information). It could be observed that the marking of the scale through A0, A1, A2, and A3 could be clearly seen. There was no distortion of the markings on the scale. However, in the case of A4, the markings appeared slightly hazy. This is an indication of reduced transparency when dp-CNC is incorporated within the films in higher quantity.

Nonetheless, researchers are extensively studying the microstructures of polymer matrices. This is because an alteration in the microstructure can alter the properties, including physicochemical and mechanical properties, of the polymer matrices. Even a slight change in the microstructure can greatly alter the properties of the polymer matrices. The polymer scientists have reported adding two or more polymers for tailoring the properties. In this regard, PVA has been blended with various polysaccharides, including GG (pristine or modified), by many researchers [23,24]. In many studies, PVA has been blended together with GG and other polymers (e.g., chitosan, tamarind seed kernel powder, κ-carrageenan, cellulose, etc.) [25,26,27,28]. Further, the properties of the blends of PVA and GG have also been tailored using nanoparticles [26]. In all of the papers, it has been reported that the addition of GG, other polymers, and nanoparticles have considerably affected the properties of the PVA films.

### 3.2. Spectroscopic Analyses

**Figure 3:**
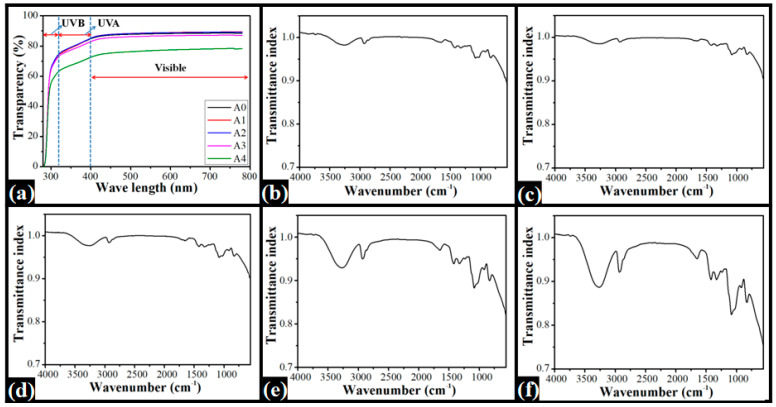
Spectroscopic analyses of the films: (**a**) UV-Vis spectra of the prepared films; (**b**–**f**) FTIR spectra of the films ((**b**) A0, (**c**) A1, (**d**) A2, (**e**) A3, and (**f**) A4).

The UV-Vis spectra of the films are shown in Figure 3a. From the spectral profiles, it can be seen that at ~280 nm (start region of UVB radiation), the absorption by all the films was very high, thereby resulting in near-zero transparency. However, by the wavelength of 315 nm (end region of UVB radiation), there was a considerable increase in the transparency values of the films [29]. The A0, A1, A2, and A3 films showed similar transparency values ~73%, but the A4 films showed significantly lower transparency (~62%). In the UVC region, there was an increase in the transparency of the films throughout the radiation wavelengths [30]. The A0, A1, and A2 films showed similar transparency values, higher than A3 and A4 films. The transparency of the A3 film was slightly lower than the transparency of the A0, A1, and A2 films. Nevertheless, the transparency of the A4 films was significantly lower than the rest of the films. Further, in the visible region, the transparency values remained constant. There was not a considerable variation in the transparency values of the films from the transparency values at 400 nm. The spectra in the visible region are concurrent with the results from the apparent transparency. In the apparent transparency test, it was found that there was a considerable distortion in the markings of the scale when overlayed with A4 film.

Figure 3b–f represent the FTIR spectra of the films. The control (A0) film showed the absorption bands at ~2929 cm^−1^, ~2861 cm^−1^, ~1649 cm^−1^, ~1420 cm^−1^, ~1329 cm^−1^, ~1086 cm^−1^, ~1028 cm^−1^, ~919 cm^−1^, and ~824 cm^−1^. The C–H stretching vibrations in the alkyl groups in PVA and guar gum can be attributed to the appearance of the peak at 2929 cm^−1^. The CH_2_ bending vibrations in PVA and guar gum are responsible for the band at 1651 cm^−1^. The absorption band at 1420.2 cm^−1^, 1086.2 cm^−1^, 919.3 cm^−1^, and 828.6 cm^−1^ were attributed to C–H wagging vibrations in the -CH_2_ group, C–O, CH_2_ stretching, and C–C stretching vibrations, respectively [31,32]. All the films showed the presence of all the peaks at similar locations. This observation suggested that the interactions in A0 and dp-CNC films were similar. The addition of dp-CNCs in the films did not significantly affect the nature of the interactions.

**Figure 4:**
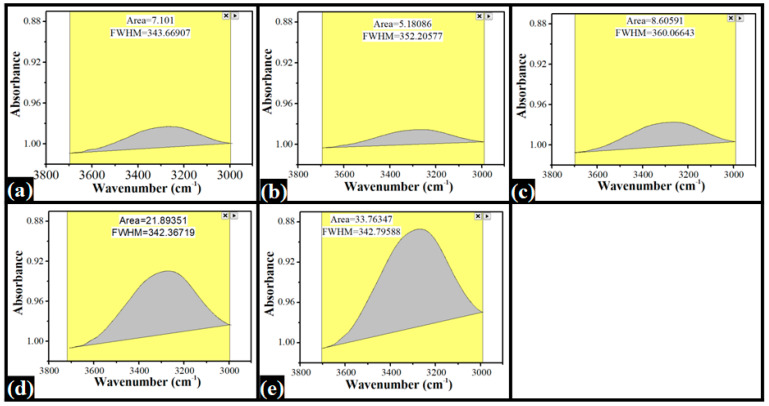
The area under the broad absorption band (3700 cm^−1^ and 2997 cm^−1^) of the films: (**a**) A0, (**b**) A1, (**c**) A2, (**d**) A3, and (**e**) A4.

Further, there was a broad absorption band in the wavenumber ranges of 3700 cm^−1^ and 2997 cm^−1^ in all of the films (Figure 4). This broad absorption band can be explained by the hydrogen bonding and -OH stretching vibrations within the components of the films [33]. The area under the peak (AUP) of this broad absorption band is a marker of hydrogen bonding. The AUP of the control film (A0) was 7.10. The AUP of A1 film, having the lowest amount of dp-CNCs, decreased to 5.18. However, with a further increase in dp-CNC content within the films A2, A3, and A4, the AUP was increased to 8.61, 21.89, and 33.76, respectively. The increase in AUP could be due to the increase in the intermolecular and intramolecular hydrogen bonds within the film components [34]. A significant increase in the hydrogen bonding may be attributed to the appearance of microcracks in A3 and A4 films.

### 3.3. XRD Study

**Figure 5:**
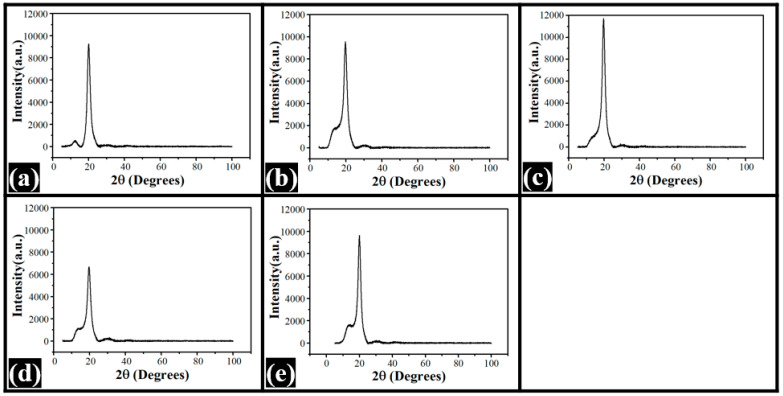
XRD diffractograms of the films: (**a**) A0, (**b**) A1, (**c**) A2, (**d**) A3, and (**e**) A4.

The diffractograms of the films are provided in Figure 5. The control (A0) films showed two major peaks at 12.50° and 20.05° 2θ. The former was a broad peak and had a lower intensity (565.34 arbitrary units (a.u.)). This peak could be associated with the amorphous region within the films. The second peak was a sharp peak, with an intensity of 9271.68 a.u., and could be attributed to the crystalline region. From the intensity values of these peaks, percentage crystallinity (%C) was calculated as per Equation (1) [35]. The %C was found to be 93.90%.

(1) $$\%C=\frac{I_{c}-I_{a}}{I_{c}}\times 100$$

where %C is the percentage of crystallinity, I_a_ is the intensity of the amorphous region, and I_c_ is the intensity of the crystalline region. 

The diffractograms of the dp-CNC-loaded films appeared similar in nature. These films also showed amorphous and crystalline peaks, as seen in A0. However, both of the bands were conjoined, unlike in A0. The amorphous peaks of A1, A2, A3, and A4 were located at 13.64°, 14.7°, 13.85°, and 13.62° 2θ, respectively. It can be observed here that the position of the amorphous peak of A2 was at a higher 2θ value than A1. Thereafter, there was a decrement in the 2θ values in A3 and A4. The corresponding peak intensities were 1787.38, 1026.56, 1133.27, and 1645.09 a.u. As the dp-CNC amount was increased in A2, there was a decrease in the intensity values. In fact, the intensity value of the amorphous peak of A2 was the lowest. After that, the intensity values of the amorphous peaks increased in a concentration-dependent manner. The crystalline peaks in the dp-CNC-loaded films were present at 19.66°, 19.56°, 19.72° and 19.96° 2θ values [35]. The position of the crystalline peak of A2 was at the lowest position. An increment in the dp-CNC content resulted in the increment in the 2θ values of A3 and A4, respectively. The intensity of the crystalline peaks of A1, A2, A3, and A4 were 9575.19, 11,732.9, 6692.58, and 9642.71 a.u., respectively. The results suggested that the highest intensity of the crystalline peak was exhibited by A2. However, no trend regarding the variation in the intensity of the crystalline peaks could be deciphered. %C was calculated from the intensity values of the amorphous and crystalline peaks [35]. The %C of the dp-CNC-loaded films was lower than the control film (93.90%). Among the dp-CNC-loaded films, the %C values of A1, A2, A3, and A4 were found to be 81.33, 91.25, 83.07, and 82.94, respectively. The %C of A2 was highest, followed by A3, A4, and A1.

### 3.4. Mechanical Study

**Figure 6:**
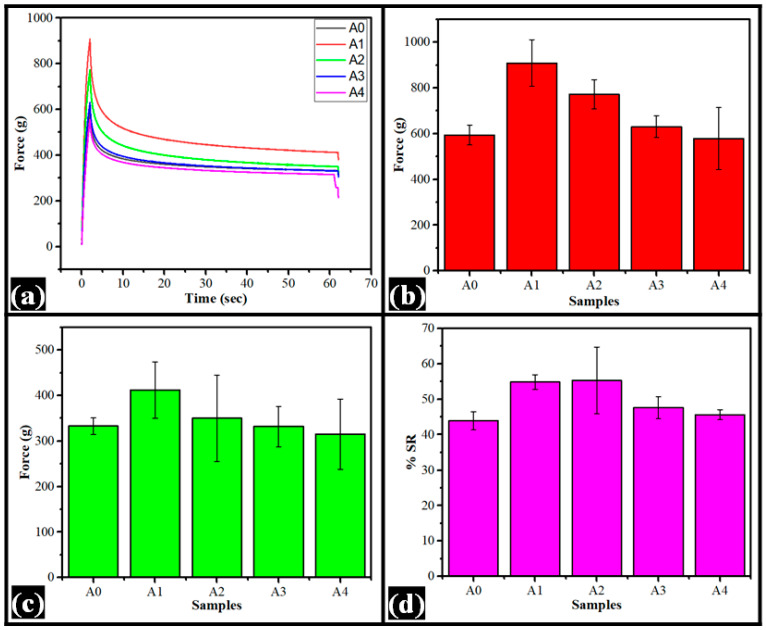
Mechanical properties of the films: (**a**) stress relaxation profiles, (**b**) F_0_ values, (**c**) F_60_ values, and (**d**) percent stress relaxation profiles.

The mechanical properties of the films were analyzed by the stress relaxation (SR) study. This method allows the researchers to analyze the changes in the polymer architecture when they are subjected to a constant strain. The SR profiles are shown in Figure 6a. With the increase in the strain, there was an increase in the force values. The force values reached the maximum (F_0_), related to firmness, when the films were stretched to the maximum extent (2 mm) (Figure 6b). The F_0_ value of A0 was 593.63 ± 43.33 g. The inclusion of dp-CNCs into the films significantly increased the firmness of A1 (F_0_ = 908.51 ± 102.26 g), explained by the reinforcing effect of dp-CNC. A further increase in the dp-CNC content correspondingly reduced the F_0_ value of the films. The F_0_ values of A1 and A2 were significantly different from the control film (*p* < 0.05). The variations in the F_0_ values of A3 and A4 were statistically similar to the control film (*p* > 0.05). Among the dp-CNC-loaded films, the F_0_ values of A1–A3, A1–A4, and A2–A3 were statistically different (*p* < 0.05).

The relaxed force values (F_60_) of the films showed a similar trend as that of F_0_ values (Figure 6c). However, the differences in the F_60_ values of the films were statistically insignificant (*p* > 0.05). Subsequently, percentage SR (%SR) values were calculated using the F_0_ and F_60_ values (Equation (2)) [36]. The %SR of A_0_ was 43.87 ± 2.51 g, which was increased in A1 (54.81 ± 2.09 g) and A2 (55.26 ± 9.42 g) (Figure 6d). The %SR values of A1 and A2 were statistically similar. Even though the %SR values of A0 and A1 were significantly different (*p* < 0.05), the %SR values of A0 and A2 were similar (*p* > 0.05). A consequent increase in the dp-CNCs in the films reduced the %SR values in A3 (47.54 ± 3.08 g) and A4 (45.61 ± 1.40 g), respectively. Among the dp-CNC-containing films, the reduction in the %SR values of A3 and A4 were statistically different (*p* < 0.05). It can be observed that the average %SR values of A1 and A2 were greater than 50%. This is suggestive of a more fluidic component in the films, compared with the others, which are predominantly elastic [37].

(2) $$\%\mathrm{SR}=\frac{F_{0}-F_{60}}{F_{0}}\times 100$$

where %SR is percentage stress relaxation, F_0_ is maximum force attained at the maximum strain, and F_60_ is the force at the end of the relaxation period.

In gist, it can be observed that A1 showed higher firmness over the other films due to the reinforcing effect of dp-CNCs. However, it also showed the most increased fluidity, compared with others. Such property can help improve the endurance of the films.

### 3.5. Drug Release Study

**Figure 7:**
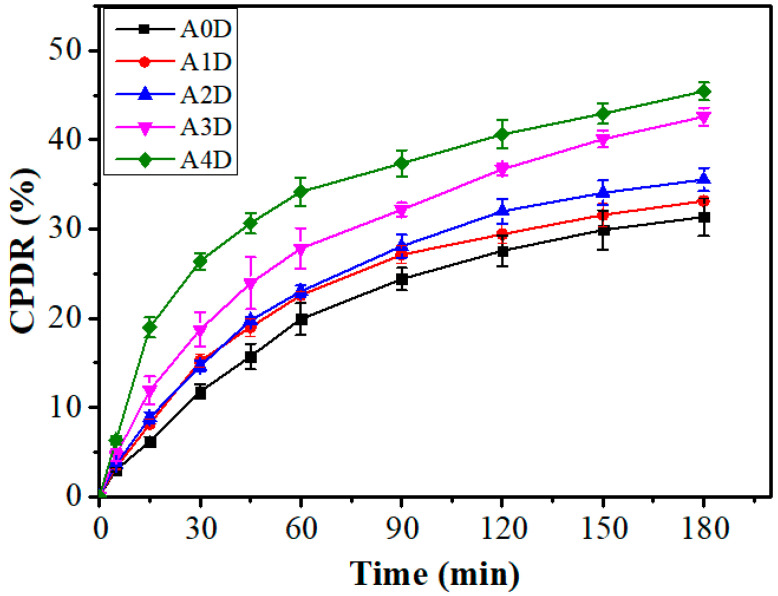
Cumulative percent drug release profiles: A0D, without dp-CNC, with 400 mg of drug; A1D, 2.5 mg dp-CNC, with 400 mg of drug; A2D, 5 mg dp-CNC, with 400 mg of drug; A3D, 7.5 mg dp-CNC, with 400 mg of drug; A4D, 10 mg of dp-CNC, with 400 mg of drug.

The moxifloxacin drug release profiles from the films are provided in Figure 7. It can be observed that there were corresponding increases in the cumulative percent drug release (CPDR) values as time progressed. However, the CPDR vs. time plot was not a linear plot [29]. An increase in the dp-CNC content in the films correspondingly improved the CPDR values. This may be due to the increase in the hydrophilicity with the increase in the dp-CNC content. The CPDR value of A0D, A1D, A2D, A3D, and A4D at the end of the study was 31.36 ± 2.15%, 33.17 ± 0.35%, 35.56 ± 1.31%, 42.60 ± 1.01%, and 45.45 ± 1.01%, respectively. Even though the average CPDR values of A1D and A2D were higher than the CPDR value of A0D, the values were not statistically significant (*p* > 0.05). The CPDR values of A3D and A4D were significantly higher than A0D (*p* < 0.05). Among the dp-CNC containing films, except the CPDR values of A1D and A2D that were similarly valued (*p* > 0.05), all other CPDR values were significantly different (*p* < 0.05). In gist, the release of the drug from the films was not affected significantly, compared with the control, in which the dp-CNC content was on a lower side. Nevertheless, when the dp-CNC content was on a higher side, the release of the drug was considerably increased. 

## 4. Conclusions

In the current study, the synthesis of poly(vinyl alcohol)/guar-gum-based phase-separated film was discussed, which was confirmed by the bright-field and polarizing microscopy techniques. The phase-separated film thus formed was incorporated with dp-CNC as the reinforcing agent. The micrographs suggested the presence of two distinct phases within the films. The appearance of such microarchitectures can be attributed to the formation of phase-separated films. It was found that at the lowest dp-CNC content, dp-CNCs acted as reinforcing agents. Interestingly, the hydrogen bonding was lowest in this film. An increase in the dp-CNC content significantly improved the hydrogen bonding. However, the mechanical properties of the films were compromised significantly in A2, A3, and A4, compared with A1. In fact, in A3 and A4, the hydrogen bonds were so strong that they formed microcracks. These microcracks can be explained by the decrease in the mechanical properties of the A3 and A4 films. Lastly, it was found that the developed films were suitable as delivery vehicles for Moxifloxacin. In the future, research with other types of drugs will be performed. Additionally, the biocompatibility of the films will be ascertained using in vitro cell culture studies and in vivo animal studies.

## Data Availability

Data are contained within the article.

## Supplementary Materials

The following supporting information can be downloaded at: https://www.mdpi.com/article/10.3390/nano12071104/s1, Figure S1: The prepared phase-separated films placed over scales to analyze morphology and transparency.

## References

[1] Yadav I., Rathnam V.S.S., Yogalakshmi Y., Chakraborty S., Banerjee I., Anis A., Pal K. (2017). Synthesis and characterization of polyvinyl alcohol-carboxymethyl tamarind gum based composite films. Carbohydr. Polym..

[2] Yadav I., Nayak S.K., Rathnam V.S.S., Banerjee I., Ray S.S., Anis A., Pal K. (2018). Reinforcing effect of graphene oxide reinforcement on the properties of poly (vinyl alcohol) and carboxymethyl tamarind gum based phase-separated film. J. Mech. Behav. Biomed. Mater..

[3] Ali H.E., Algarni H., Khairy Y. (2020). Influence of cobalt-metal concentration on the microstructure and optical limiting properties of PVA. Opt. Mater..

[4] Arefian M., Hojjati M., Tajzad I., Mokhtarzade A., Mazhar M., Jamavari A. (2020). A review of Polyvinyl alcohol/Carboxymethyl cellulose (PVA/CMC) composites for various applications. J. Compos. Compd..

[5] Zulkiflee I., Fauzi M.B. (2021). Gelatin-Polyvinyl Alcohol Film for Tissue Engineering: A Concise Review. Biomedicines.

[6] Rezaei F.S., Sharifianjazi F., Esmaeilkhanian A., Salehi E. (2021). Chitosan films and scaffolds for regenerative medicine applications: A review. Carbohydr. Polym..

[7] Bi S., Wang P., Hu S., Li S., Pang J., Zhou Z., Sun G., Huang L., Cheng X., Xing S. (2019). Construction of physical-crosslink chitosan/PVA double-network hydrogel with surface mineralization for bone repair. Carbohydr. Polym..

[8] Feng R., Fu R., Duan Z., Zhu C., Ma X., Fan D., Li X. (2018). Preparation of sponge-like macroporous PVA hydrogels via n-HA enhanced phase separation and their potential as wound dressing. J. Biomater. Sci. Polym. Ed..

[9] Gadhave R.V., Mahanwar P.A., Gadekar P.T. (2019). Effect of glutaraldehyde on thermal and mechanical properties of starch and polyvinyl alcohol blends. Des. Monomers Polym..

[10] Sarwar M.S., Niazi M.B.K., Jahan Z., Ahmad T., Hussain A. (2018). Preparation and characterization of PVA/nanocellulose/Ag nanocomposite films for antimicrobial food packaging. Carbohydr. Polym..

[11] Zhang H., Zhang J. (2020). The preparation of novel polyvinyl alcohol (PVA)-based nanoparticle/carbon nanotubes (PNP/CNTs) aerogel for solvents adsorption application. J. Colloid Interface Sci..

[12] Rolim W.R., Pieretti J.C., Renó D.B.L., Lima B.A., Nascimento M.N.H., Ambrosio F.N., Lombello C.B., Brocchi M., de Souza A.C.S., Seabra A.B. (2019). Antimicrobial activity and cytotoxicity to tumor cells of nitric oxide donor and silver nanoparticles containing PVA/PEG films for topical applications. ACS Appl. Mater. Interfaces.

[13] Maheshwari T., Tamilarasan K., Selvasekarapandian S., Chitra R., Kiruthika S. (2021). Investigation of blend biopolymer electrolytes based on Dextran-PVA with ammonium thiocyanate. J. Solid State Electrochem..

[14] Samadi N., Sabzi M., Babaahmadi M. (2018). Self-healing and tough hydrogels with physically cross-linked triple networks based on Agar/PVA/Graphene. Int. J. Biol. Macromol..

[15] Perez-Puyana V., Jiménez-Rosado M., Romero A., Guerrero A. (2018). Development of PVA/gelatin nanofibrous scaffolds for Tissue Engineering via electrospinning. Mater. Res. Express.

[16] Jadbabaei S., Kolahdoozan M., Naeimi F., Ebadi-Dehaghani H. (2021). Preparation and characterization of sodium alginate–PVA polymeric scaffolds by electrospinning method for skin tissue engineering applications. RSC Adv..

[17] Mombini S., Mohammadnejad J., Bakhshandeh B., Narmani A., Nourmohammadi J., Vahdat S., Zirak S. (2019). Chitosan-PVA-CNT nanofibers as electrically conductive scaffolds for cardiovascular tissue engineering. Int. J. Biol. Macromol..

[18] Kraskouski A., Hileuskaya K., Kulikouskaya V., Kabanava V., Agabekov V., Pinchuk S., Vasilevich I., Volotovski I., Kuznetsova T., Lapitskaya V. (2021). Polyvinyl alcohol and pectin blended films: Preparation, characterization, and mesenchymal stem cells attachment. J. Biomed. Mater. Res. Part A.

[19] Verma D., Sharma S.K. (2021). Recent advances in guar gum based drug delivery systems and their administrative routes. Int. J. Biol. Macromol..

[20] Kumar B., Murali A., Bharath A.B., Giri S. (2019). Guar gum modified upconversion nanocomposites for colorectal cancer treatment through enzyme-responsive drug release and NIR-triggered photodynamic therapy. Nanotechnology.

[21] Das S., Ghosh B., Sarkar K. (2022). Nanocellulose as sustainable biomaterials for drug delivery. Sens. Int..

[22] Shaikh H.M., Anis A., Poulose A.M., Al-Zahrani S.M., Madhar N.A., Alhamidi A., Alam M.A. (2021). Isolation and Characterization of Alpha and Nanocrystalline Cellulose from Date Palm (Phoenix dactylifera L.) Trunk Mesh. Polymers.

[23] Dalei G., Das S., Das S.P. (2021). Low-pressure nitrogen and ammonia plasma treatment on carboxymethyl guar gum/PVA hydrogels: Impact on drug delivery, biocompatibility and biodegradability. Int. J. Polym. Mater. Polym. Biomater..

[24] Gupta A.P., Arora G. (2011). Preparation and characterization of guar-gum/polyvinylalcohol blend films. J. Mater. Sci. Eng. B.

[25] Iqbal D.N., Shafiq S., Khan S.M., Ibrahim S.M., Abubshait S.A., Nazir A., Abbas M., Iqbal M. (2020). Novel chitosan/guar gum/PVA hydrogel: Preparation, characterization and antimicrobial activity evaluation. Int. J. Biol. Macromol..

[26] Gasti T., Hiremani V.D., Kesti S.S., Vanjeri V.N., Goudar N., Masti S.P., Thimmappa S.C., Chougale R.B. (2021). Physicochemical and Antibacterial Evaluation of Poly (Vinyl Alcohol)/Guar Gum/Silver Nanocomposite Films for Food Packaging Applications. J. Polym. Environ..

[27] Iqbal D.N., Tariq M., Khan S.M., Gull N., Iqbal S.S., Aziz A., Nazir A., Iqbal M. (2020). Synthesis and characterization of chitosan and guar gum based ternary blends with polyvinyl alcohol. Int. J. Biol. Macromol..

[28] Patil C.M., Borse A.U., Meshram J.S. (2018). Ionic liquid: Green solvent for the synthesis of cellulose/guar gum/PVA biocomposite. Green Mater..

[29] Qureshi D., Sahoo A., Mohanty B., Anis A., Kulikouskaya V., Hileuskaya K., Agabekov V., Sarkar P., Ray S.S., Maji S. (2021). Fabrication and Characterization of Poly (vinyl alcohol) and Chitosan Oligosaccharide-Based Blend Films. Gels.

[30] Qureshi D., Pattanaik S., Mohanty B., Anis A., Kulikouskaya V., Hileuskaya K., Agabekov V., Sarkar P., Maji S., Pal K. (2021). Preparation of novel poly(vinyl alcohol)/chitosan lactate-based phase-separated composite films for UV-shielding and drug delivery applications. Polym. Bull..

[31] Mathew S., Jayakumar A., Kumar V.P., Mathew J., Radhakrishnan E.K. (2019). One-step synthesis of eco-friendly boiled rice starch blended polyvinyl alcohol bionanocomposite films decorated with in situ generated silver nanoparticles for food packaging purpose. Int. J. Biol. Macromol..

[32] Singh S., Gaikwad K.K., Lee Y.S. (2018). Antimicrobial and antioxidant properties of polyvinyl alcohol bio composite films containing seaweed extracted cellulose nano-crystal and basil leaves extract. Int. J. Biol. Macromol..

[33] Hendrawan H., Khoerunnisa F., Sonjaya Y., Putri A.D. Poly (vinyl alcohol)/glutaraldehyde/Premna oblongifolia merr extract hydrogel for controlled-release and water absorption application. Proceedings of the IOP Conference Series: Materials Science and Engineering.

[34] Nizan N.S.N.H., Zulkifli F.H. (2020). Reinforcement of hydroxyethyl cellulose/poly (vinyl alcohol) with cellulose nanocrystal as a bone tissue engineering scaffold. J. Polym. Res..

[35] Sagiri S.S., Singh V.K., Pal K., Banerjee I., Basak P. (2015). Stearic acid based oleogels: A study on the molecular, thermal and mechanical properties. Mater. Sci. Eng. C.

[36] Das P., Qureshi D., Paul S., Mohanty B., Anis A., Verma S., Wilczyński S., Pal K. (2021). Effect of sorbitan monopalmitate on the polymorphic transitions and physicochemical properties of mango butter. Food Chem..

[37] Bharti D., Kim D., Cerqueira M.A., Mohanty B., Habibullah S., Banerjee I., Pal K. (2021). Effect of Biodegradable Hydrophilic and Hydrophobic Emulsifiers on the Oleogels Containing Sunflower Wax and Sunflower Oil. Gels.

