# Supplementary material for: Date-Palm-Derived Cellulose Nanocrystals as Reinforcing Agents for Poly(vinyl alcohol)/Guar-Gum-Based Phase-Separated Composite Films
Source: Nanomaterials (Basel). 2022 Mar 27;12(7):1104. doi: 10.3390/nano12071104 (PMC9000832; doi:10.3390/nano12071104)
Supplement: Supplementary file 1 [file nanomaterials-12-01104-s001.zip › nanomaterials-1627629-supplementary.pdf]

Supplementary Information

Figure. S1. The prepared phase-separated films placed over scales to analyze morphology and transparency

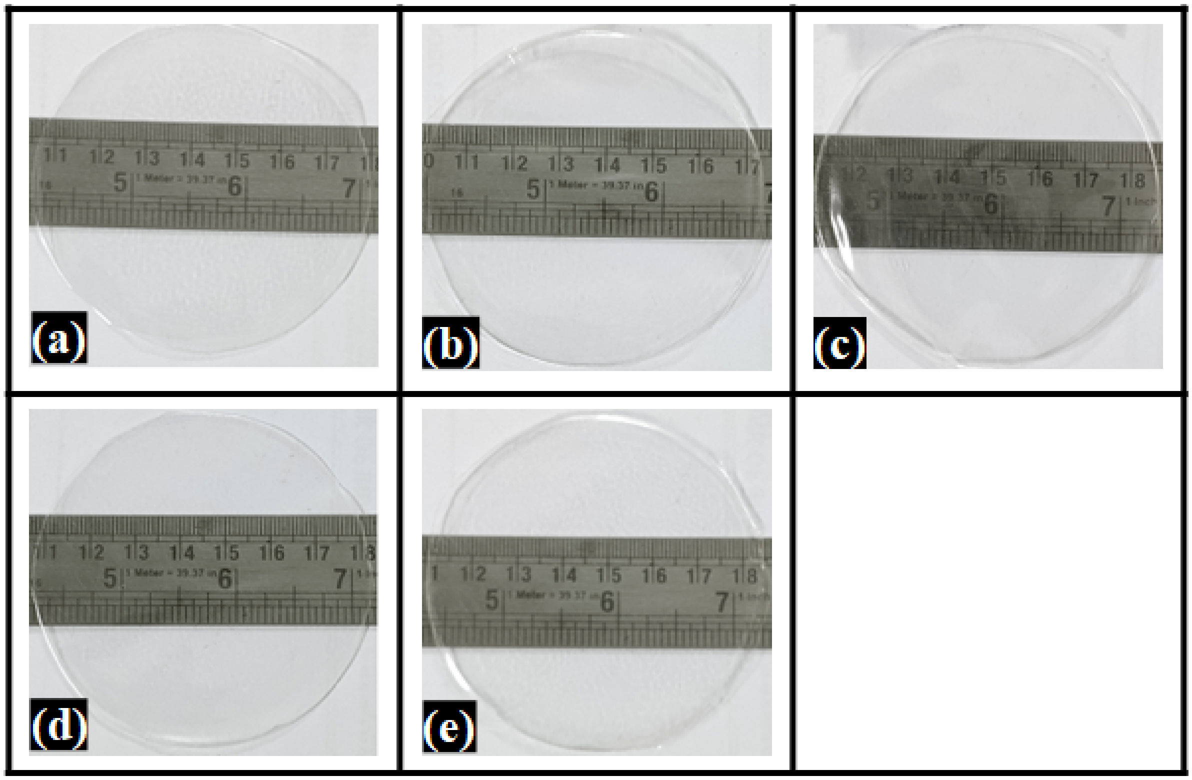

Figure.S1. Phase separated films (a) A0, (b) A1, (c) A2, (d) A3, and (e) A4.
